# A Nomogram for Predicting Lung Metastasis in Papillary Thyroid Cancer Patients Aged Less Than 55 Years

**DOI:** 10.3389/fendo.2025.1689674

**Published:** 2025-11-11

**Authors:** Huiyun Yang, Qiuqin Qian, Yanling Li, Wenjie Pan, Jing Peng, Zhenyu Zou, Haiqing Zhu, Feng Shi

**Affiliations:** 1 Department of Nuclear Medicine, The Affiliated Cancer Hospital of Xiangya School of Medicine, Central South University, Hunan Cancer Hospital, Changsha, China; 2 Department of Radiology, Second Affiliated Hospital of Hunan University of Chinese Medicine, Changsha, Hunan, China

**Keywords:** thyroid cancer, lung metastasis, nomogram, next-generation sequencing, prognosis

## Abstract

**Highlights:**

A nomogram predicting lung metastasis in patients with papillary thyroid cancer was developed and validated. Our study focused on patients under 55 years old, revealing the genetic factors linked to lung metastasis in this younger group. Young patients with the BRAF V600E mutation are less likely to develop lung metastasis.

**Objective:**

To develop and validate a nomogram for predicting the risk of lung metastasis in patients under 55 years old with papillary thyroid cancer (PTC).

**Methods:**

A total of 243 patients under 55 years old with PTC were retrospectively collected from January 2017 to June 2020 and randomly divided into a training group (n = 170) and a validation group (n = 73) in a 7:3 ratio. Genetic testing data and clinical information were compiled, and univariate and multivariate binary logistic regression analyses were performed. Based on the results, a nomogram predicting the risk of lung metastasis was constructed using the training cohort. The nomogram’s performance was assessed using calibration curves, decision curve analysis (DCA), the concordance index (C-index), and the area under the receiver operating characteristic (ROC) curve (AUC) in both the training and validation groups.

**Results:**

T stage, unilateral thyroid involvement, TERT mutation, and BRAF mutation were identified as independent prognostic factors and were used to construct the nomogram. The C-index of the model was 0.89 in the training group and 0.88 in the validation group. The AUC, DCA, and calibration curves demonstrated favorable predictive accuracy. Using a cut-off value of 0.229, the nomogram achieved a sensitivity of 0.829 and a specificity of 0.793.

**Conclusion:**

A nomogram with strong predictive performance has been successfully developed and validated, which may assist clinicians in estimating the risk of lung metastasis in young patients with PTC.

## Introduction

Papillary thyroid cancer (PTC) is the most common endocrine malignancy of the head and neck. Differentiated thyroid cancer accounts for over 90% of thyroid cancer cases. The standard treatment typically involves radioactive iodine-131 therapy or active surveillance following surgery. Although most patients have a favorable prognosis, 10-15% still develop distant metastases, and the 10-year overall survival rate in this subgroup is only about 40% (1, 2). Therefore, identifying factors associated with distant metastasis is critical for the early recognition of high-risk patients and the timely initiation of individualized treatment strategies.

Previous studies have investigated risk factors for distant metastasis in differentiated thyroid cancer. Khan U et al. (3) reported that tumor size ≥2 cm, nodal metastasis, and histological subtype are significantly associated with distant spread. Other researchers have identified tumor size, age, surgical intervention, N stage, T stage, and pathological type as independent predictors of distant metastasis in female patients with differentiated thyroid cancer (4). In recent years, the role of genetic mutations in metastatic risk has gained increasing attention. Gene fusions involving *RET*, *ALK*, or *NTRK1*, among others, have been shown to correlate with metastatic potential in thyroid cancer (5). However, few studies have integrated clinical features with genetic testing data to predict the risk of distant metastasis in these patients.

The lungs are the most common site of distant metastasis in PTC, accounting for approximately 45% of all metastatic cases (6). Pulmonary metastasis is also a leading cause of mortality in patients with PTC (7). Hence, early identification of individuals at risk for lung metastasis is essential to improve outcomes through targeted and timely intervention. While metastasis in thyroid cancer has been associated with age (4), lung metastases can still occur in younger patients under 55 years of age. Therefore, this study aims to develop a nomogram that combines clinical characteristics with genetic testing results to predict the likelihood of lung metastasis in patients under 55 years old with PTC.

## Patients and methods

This study was approved by the Ethics Committee of the Affiliated Cancer Hospital of Xiangya School of Medicine, Central South University/Hunan Cancer Hospital (Approval No. KYJJ-2020-195).

### Study population

A total of 243 patients under 55 years old with PTC were selected from the Affiliated Cancer Hospital of Xiangya School of Medicine between January 2017 and June 2020 and followed up until July 2023. The inclusion criteria were as follows: (I) postoperative histopathological confirmation of PTC; (II) age <55 years; (III) availability of pathological tissue and complete clinical data; (IV) no evidence of metastasis at the time of initial diagnosis; and (V) development of pulmonary metastases only during the follow-up period. For patients diagnosed solely with “lung metastasis,” at least one of the following criteria was required to confirm the diagnosis (1): pulmonary pathology confirmed independently by two pathologists; (2) radioactive iodine uptake in the lungs; (3) positive findings on positron emission tomography-computed tomography (PET-CT); or (4) chest computed tomography (CT) findings reviewed and confirmed by two radiologists. The exclusion criteria were: (I) the presence of other primary tumors in addition to PTC; (II) metastasis to organs other than the lungs; and (III) other pathological subtypes of thyroid cancer. “unilateral thyroid involvement” is determined based on postoperative histopathological reports. It is defined as: the primary tumor is confined to a single thyroid lobe, with no evidence of tumor involvement in the contralateral lobe on pathological examination.

### Genetic testing

All patients underwent whole-genome sequencing using next-generation sequencing (NGS) with the Geneseeq Prime™ 425-gene panel. The specific genes analyzed are listed in **Supplementary Table 1**. The sequencing procedure was as follows: DNA was extracted from pathological tissue samples. The extracted DNA was then end-repaired, and poly(A) tails were added. Illumina sequencing adapters were ligated to both ends of the DNA fragments, followed by library construction through iterative optimization of conditions. Custom-designed DNA probes targeting oncogenes were synthesized to enrich the library for relevant genes.

Enriched libraries of tumor tissue oncogene exons and negative controls were prepared, pooled based on sequencing throughput, and subjected to high-throughput sequencing using the PE75 kit (Illumina, California, USA). Bioinformatics analysis was subsequently performed to obtain comprehensive mutation data, including single nucleotide variants (SNVs), gene fusions, amplifications, deletions, and insertions in tumor tissues.

### Construction of the nomogram

The data were organized, and univariate binary logistic regression analysis was performed to identify variables with *P* < 0.05. These significant variables were then included in a multivariate binary logistic regression analysis with stepwise selection to identify independent predictors (*P* < 0.05) for constructing the nomogram. Statistical analyses were conducted using SPSS software and R language.

The Hosmer-Lemeshow test was used to evaluate the goodness of fit between the observed outcomes and the nomogram’s predictions. A calibration curve was generated to compare the predicted probability of lung metastasis with the actual observed outcomes. Decision curve analysis (DCA) was employed to assess the clinical utility and net benefit of the nomogram. The apparent performance of the nomogram is evaluated using the concordance index (C-index), and its optimistic bias is corrected through 1000 bootstrap resamples to obtain the bias-corrected C-index. The C-index and the area under the receiver operating characteristic (ROC) curve (AUC) were calculated to evaluate the nomogram’s predictive accuracy. Using SPSS software, the probability of lung metastasis was calculated for each patient in the training group. The optimal cut-off value, also known as Youden index, was determined using MedCalc software (8).

### Statistical analysis

Statistical analyses were performed using SPSS software and R version 4.3.2 (http://www.R-project.org/), with *P* < 0.05 considered statistically significant. MedCalc software (Ostend, Belgium) was used to determine the optimal cut-off value.

## Results

### Patients’ characteristics

From January 2017 to June 2020, a total of 243 patients under 55 years old with PTC were enrolled in the study and randomly assigned to a training group (n = 170) and a validation group (n = 73) in a 7:3 ratio. The clinical characteristics of the patients are summarized in **Table 1**. The median age was 36 years in the training group and 38 years in the validation group. Lung metastases were observed in 35 patients in the training group and 15 patients in the validation group. A total of 151 patients had unilateral thyroid lobe involvement. The mean maximum diameter of primary thyroid lesions was 2.3 cm in the training group and 1.8 cm in the validation group. Extraglandular invasion was observed in 51.2% of patients in the training group and 46.6% in the validation group.

**Table 1:** The characteristics of patients in the training and validation cohorts.

Characteristic	Training cohort (n=170)	Validation cohort (n=73)	P
Average age (years)	36	38	0.109
Sex			0.165
Male Female	35 (20.6%) 135 (79.4%)	21 (28.8%) 52 (71.2%)	
T* stage			0.822
T1	61 (35.9%)	31 (42.5%)	
T2	17 (10.0%)	6 (8.2%)	
T3	58 (34.1%)	22 (30.1%)	
T4	34 (20.0%)	14 (19.2%)	
N* stage			0.231
N0	12 (7.1%)	6 (8.2%)	
N1a	61 (35.9%)	18 (24.7%)	
N1b	97 (57.1%)	49 (67.1%)	
Lung metastasis			0.994
YES	35 (20.6%)	15 (20.5%)	
NO	135 (79.4%)	58 (79.5%)	
Unilateral thyroid involvement			0.104
YES	100 (58.8%)	51 (69.9%)	
NO	70 (41.2%)	22 (30.1%)	
Maximum diameter (cm)	2.3	1.8	0.002
Extra-glandular invasion			0.748
YES	87 (51.2%)	39 (46.6%)	
NO	83 (48.8%)	34 (53.4%)	
RET*			0.554
YES	23 (13.5%)	12 (85.6%)	
NO	147 (86.5%)	61 (14.4%)	
NRAS*			0.104
YES	6 (3.5%)	0 (0.0%)	
NO	164 (96.5%)	73 (100.0%)	
HRAS*			0.900
YES	2 (1.2%)	1 (1.4%)	
NO	168 (98.8%)	72 (98.6%)	
KRAS*			0.825
YES	3 (1.8%)	1 (1.4%)	
NO	167 (98.2%)	72 (98.6%)	
TERT*			0.913
YES	11 (6.5%)	5 (6.8%)	
NO	159 (93.5%)	68 (93.2%)	
BRAF* V600E			0.095
YES	97 (57.1%)	50 (68.5%)	
NO	73 (42.9%)	23 (31.5%)	
NTRK1*			0.859
YES	4 (2.4%)	2 (2.7%)	
NO	166 (97.6%)	71 (97.3%)	
PAX8*			0.126
YES	0 (0.0%)	1 (1.4%)	
NO	170 (100.0%)	72 (98.6%)	
TP53*			0.368
YES	7 (4.1%)	5 (6.8%)	
NO	163 (95.9%)	68 (93.2%)	
CTNNB1*			0.352
YES	2 (1.2%)	0 (0.0%)	
NO	168 (98.8%)	73 (100.0%)	
PIK3CA*			0.752
YES	6 (3.5%)	2 (2.7%)	
NO	164 (96.5%)	71 (97.3%)	

*T, tumor; N, lymph node; RET, rearranged during transfection; NRAS, Neuroblastoma RAS viraloncogene homolog; HRAS, Harvey Rat Sarcoma Viral Oncogene Homolog; KRAS, Kirsten Rat Sarcoma Viral Oncogene Homolog; TERT, Telomerase Reverse Transcriptase; BRAF, V-raf murine sarcoma viral oncogene homolog B; NTRK1, Neurotrophic Receptor Tyrosine Kinase 1; PAX8, Paired Box 8; TP53, Cellular Tumor Antigen P53; CTNNB1, Catenin Beta 1; PIK3CA, Phosphatidylinositol-4,5-Bisphosphate 3-Kinase Catalytic Subunit Alpha.

All patients underwent whole-genome sequencing using NGS. *BRAF* mutations were detected in 147 of 243 patients (60.4%), representing the most frequently observed mutation, followed by *RET* mutations (35/243) and *TERT* mutations (16/243). Detailed results are presented in **Table 1**.

### Identifying predictors

Univariate binary logistic regression analysis identified age, T stage, N stage, unilateral thyroid involvement, maximum tumor diameter, extra-glandular invasion, and *RET*, *TERT*, and *BRAF* gene mutations as factors significantly associated with lung metastasis in thyroid cancer. In the multivariate analysis, T stage (OR = 5.564, 95% CI: 1.769-17.502, *P* = 0.003), unilateral thyroid involvement (OR = 8.706, 95% CI: 3.002-25.248, *P* < 0.001), *TERT* mutation (OR = 7.585, 95% CI: 1.245-46.199, *P* = 0.028), and *BRAF* mutation (OR = 0.070, 95% CI: 0.022-0.225, *P* < 0.001) were confirmed as independent predictors of lung metastasis and were used to construct the nomogram in R (**Table 2**). The Hosmer-Lemeshow test yielded a *P*-value of 0.986, indicating a good model fit.

**Table 2:** Univariate and multivariate binary logistic regression analyses in the training cohort.

Factors	Univariate analysis			Multivariate analysis		
	OR	95%CI	P	OR	95%CI	P
Age (years)	0.950	0.915-0.986	0.007	–	–	–
Sex (female vs. male)	0.689	0.289-1.645	0.402	–	–	–
T stage (T1-T2 vs. T3-T4)	7.065	2.585-19.308	<0.001	5.564	1.769-17.502	0.003
N stage (N0-N1 vs. N2)	3.826	1.564-9.358	0.003	–	–	–
Unilateral thyroid involvement (Yes vs. No)	5.975	2.582-13.827	<0.001	8.706	3.002-25.248	<0.001
Maximum diameter (cm)	1.593	1.222-2.076	0.001	–	–	–
Extra-glandular invasion (No vs. Yes)	4.219	1.787-9.595	0.001	–	–	–
RET (No vs. Yes)	3.754	1.481-9.514	0.005	–	–	–
TERT (No vs. Yes)	5.379	1.536-18.836	0.009	7.585	1.245-46.199	0.028
BRAF V600E (No vs. Yes)	0.125	0.051-0.308	<0.001	0.070	0.022-0.225	<0.001
RAS(HRAS/KRAS/NRAS)	1.488	0.374-5.930	0.573			
TP53 (No vs. Yes)	0.632	0.074-5.431	0.676	–	–	–
PIK3CA (No vs. Yes)	0.765	0.086-6.765	0.809	–	–	–

*T, tumor; N, lymph node; RET, rearranged during transfection; TERT, Telomerase Reverse Transcriptase; BRAF, V-raf murine sarcoma viral oncogene homolog B; TP53, Cellular Tumor Antigen P53; PIK3CA, Phosphatidylinositol-4,5-Bisphosphate 3-Kinase Catalytic Subunit Alpha, HRAS, Harvey Rat Sarcoma viral oncogene homolog; NRAS, Neuroblastoma RAS viral oncogene homolog; KRAS, Kirsten Rat Sarcoma viral oncogene homolog.

### Construction and evaluation of nomogram

**Figure 1:**
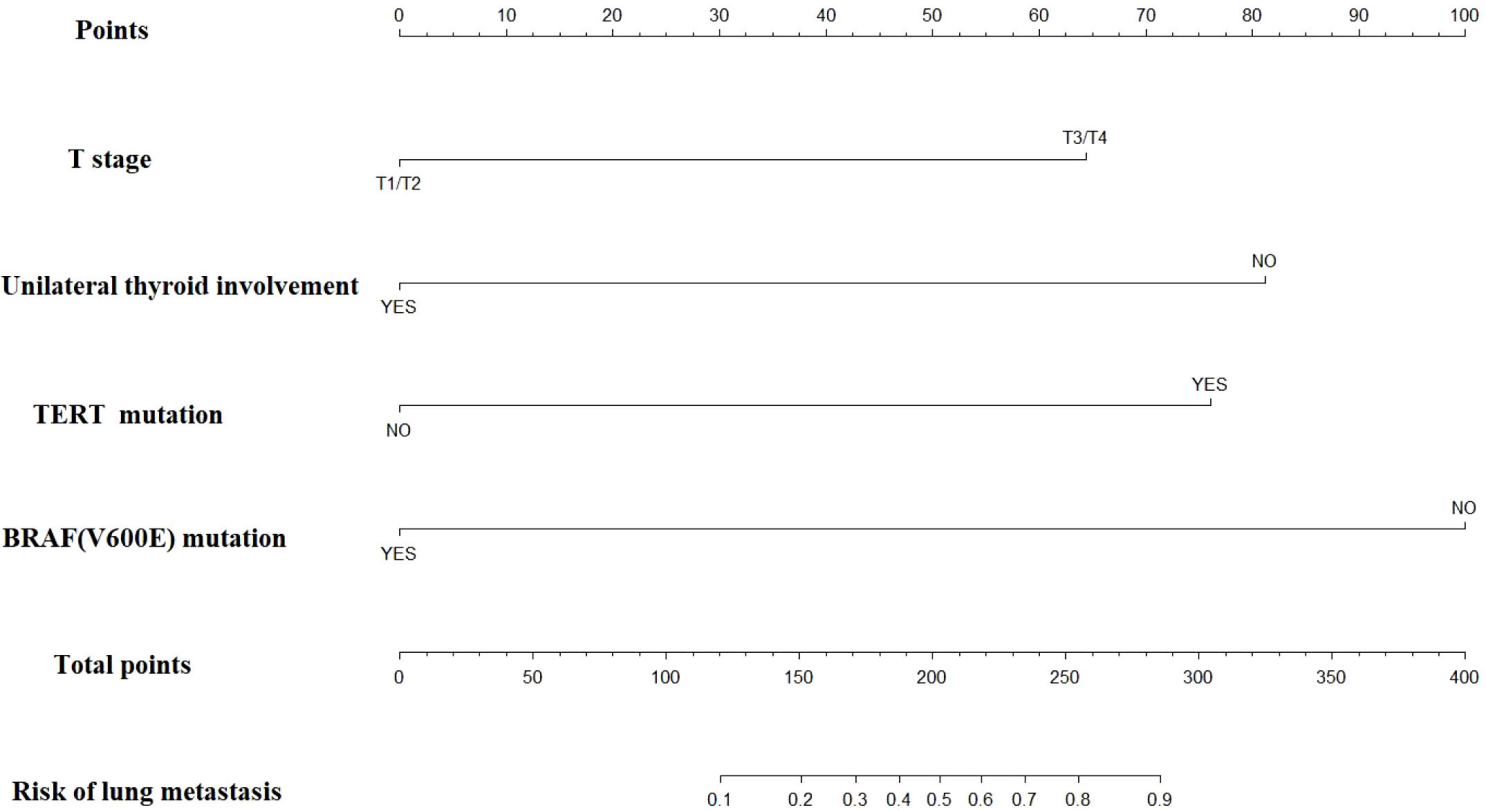
Nomogram for predicting lung metastases in patients with PTC under 55 years old.

Based on the multivariate regression analysis results, each factor was assigned a specific score. The detailed regression coefficients, odds ratios, and the score for each predictor in the final nomogram are provided in **Supplementary Table 3**. According to **Supplementary Table 3**, for young patients suspected of lung metastasis, we can assign scores based on BRAF mutation, TERT promoter mutation, unilateral thyroid involvement, and T stage. These scores are then summed to obtain the “total points” in our nomogram. A vertical line is drawn from the total points to the corresponding risk of lung metastasis, thereby determining the patient’s probability of developing lung metastasis (**Figure 1**).

**Figure 2:**
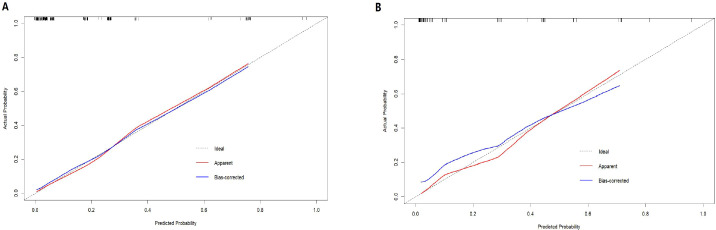
Calibration plots of the nomogram in the training **(A)** and validation **(B)** cohorts.

The nomogram demonstrated strong predictive performance, with a C-index of 0.89 in the training cohort and 0.88 in the validation cohort. Calibration curves showed good agreement between predicted and observed outcomes in both the training (**Figure 2A**) and validation (**Figure 2B**) groups. This indicates the model’s strong predictive accuracy. Such reliability enables clinicians to trust the model outputs, accurately communicate risks, and formulate appropriate treatment decisions—avoiding both overestimation that leads to overtreatment and underestimation that results in delayed intervention.

**Figure 3:**
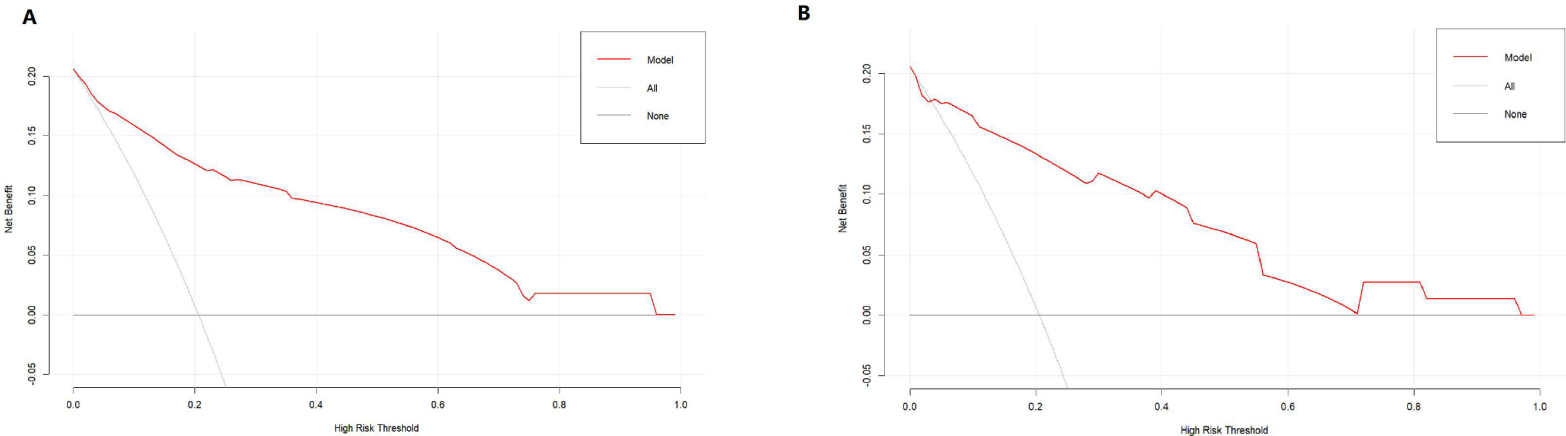
DCA of the nomogram in the training **(A)** and validation **(B)** cohorts.

**Figure 4:**
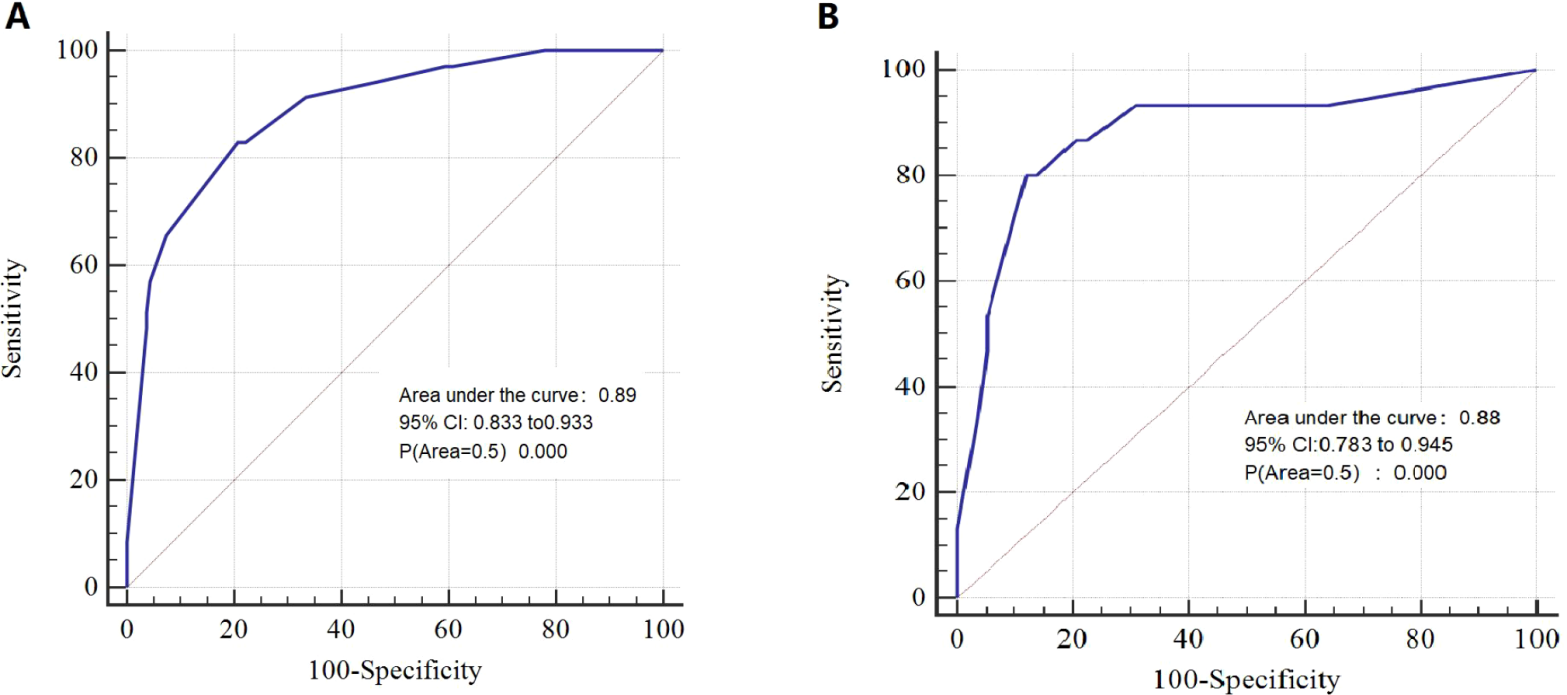
AUC of the nomogram in the training **(A)** and validation **(B)** cohorts.

**Figure 5:**
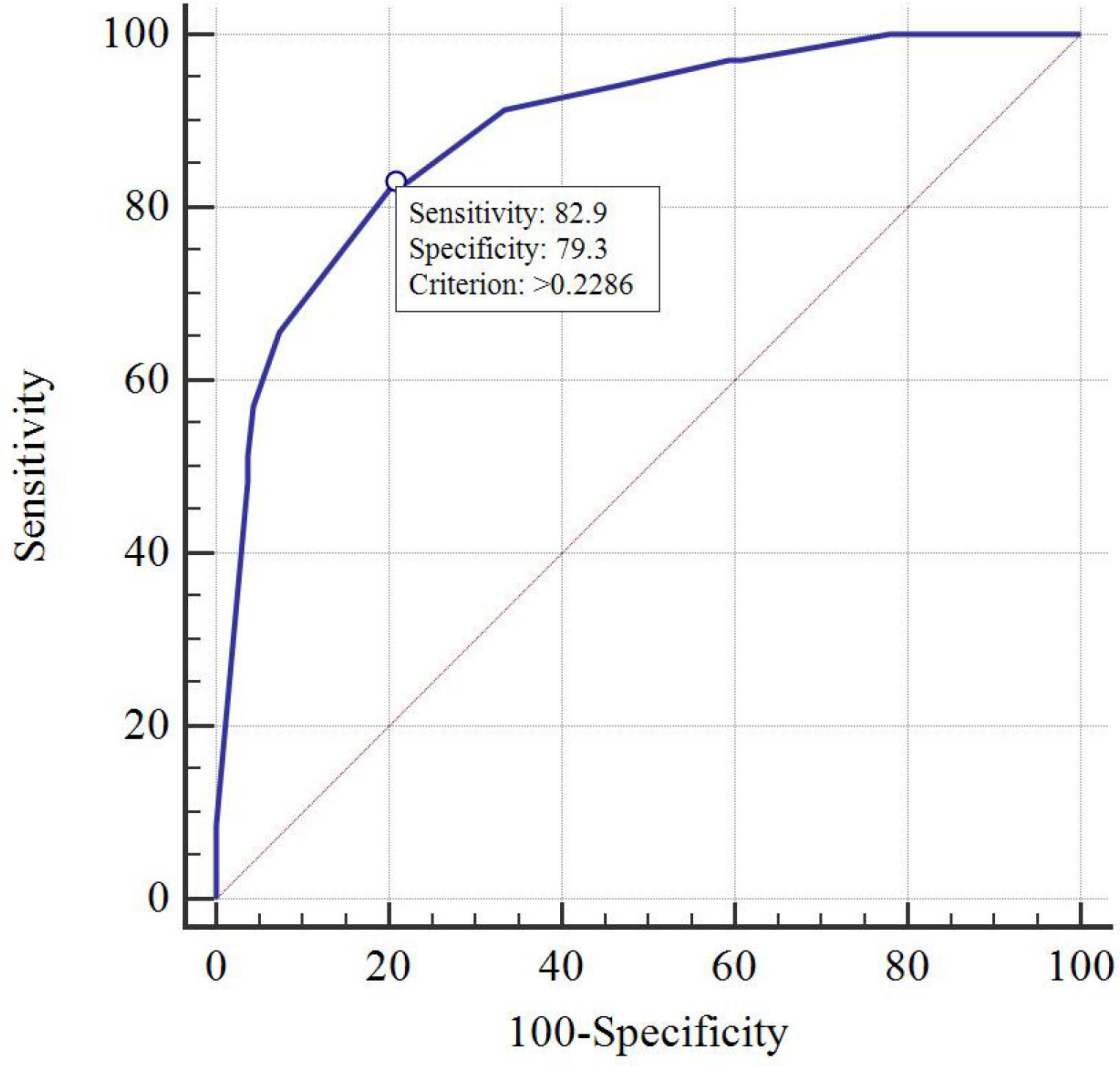
The cut-off value determined by the ROC curve in the training cohort.

DCA indicated that the nomogram has valuable clinical utility (**Figures 3A, B**). Specifically, decision analysis indicates that within a broad threshold probability range of approximately 10% to 90%, this predictive map delivers superior net benefits compared to both “all interventions” and “no interventions” strategies, which suggests that when clinicians consider intervention thresholds (such as enhanced follow-up or treatment) within this range, the nomogram can guide decision-making. The area under the ROC curve (AUC) was 0.89 for the training group and 0.88 for the validation group (**Figures 4A, B**). To assess the added value of the integrated model, we compared its performance against each independent predictor in the training cohort. As shown in **Supplementary Figure 1**, the nomogram demonstrated superior discriminatory accuracy (**Supplementary Figure 1A**) and provided a greater net benefit across a wide range of threshold probabilities (**Supplementary Figure 1B**) compared to any single variable (T stage, unilateral thyroid involvement, TERT mutation, or BRAF mutation).The analysis confirms that the integrated model outperforms any single predictor. The optimal cut-off value, defined by the Youden index, was 0.229, corresponding to a sensitivity of 82.9% and specificity of 79.3% (**Figure 5**).

## Discussion

This study developed and validated a nomogram to predict the risk of lung metastasis in patients under 55 years old with PTC by integrating clinical data and genomic sequencing information. T stage, unilateral thyroid involvement, *TERT* promoter mutation, and *BRAF* V600E mutation were identified as independent predictors of lung metastasis and incorporated into the nomogram. Patients were stratified into high-risk and low-risk groups using a risk score threshold of 0.229, with the high-risk group showing a significantly higher probability of developing lung metastases.

Several previous studies have developed nomograms to predict the prognosis of thyroid cancer (3, 4, 9), but our study differs from them in key aspects. Wen W et al. (4) constructed a nomogram including age at diagnosis, pathological type, N1 stage, T3–4 stage, thyroidectomy, and tumor size to predict distant metastasis in thyroid cancer. Peng G et al. (10) developed a nomogram based on the number of lymph node metastases, lymph node ratio, and preoperative thyroglobulin to predict survival prognosis in PTC patients. However, these models lack genetic background information. NGS has been widely recommended for molecular classification and predicting recurrence and metastasis in PTC (11). In our study, all patients underwent NGS, and our model combined clinical and genetic data to predict distant metastasis, which could reflect more genetic background information, differing from their studies.

Additionally, previous studies did not specifically focus on age stratification (4, 10). Our study targeted patients younger than 55 years old. According to the 8th edition of the AJCC staging system for thyroid cancer, patients younger than 55 years are staged differently from those 55 or older. Patients under 55 with distant metastases are classified as stage II, while those 55 or older with distant metastases are classified as stage IVB, highlighting age 55 as a critical prognostic threshold (12–14). Although younger patients generally have better prognoses, a subset still develops distant metastases and experiences poor outcomes (15, 16). Therefore, identifying risk factors for distant metastases in young patients is essential for enabling timely individualized treatment.

Third, our study specifically focuses on single-organ (lung)-specific metastasis of thyroid cancer, which is rarely addressed in previous research. The lungs are the most common and often the earliest site of metastasis in thyroid cancer patients (17–19), particularly in younger patients (15, 20). Kuang HF et al. (21) previously developed a nomogram to predict lung metastasis in young thyroid cancer patients using the SEER database; however, his target population was primarily under 19 years old, and the studied pathologies included both papillary and follicular carcinomas. Importantly, our study excluded patients with metastases to other sites, differentiating it from prior work. Given that lung metastases are typically the most frequent and initial metastatic site in thyroid cancer, this focus enhances clinical relevance.

Using our nomogram, clinicians may identify young patients at high risk for lung metastasis early and adjust clinical management accordingly. For instance, high-risk patients should undergo more frequent and stringent follow-ups, especially those presenting with multiple pulmonary nodules on imaging but without clinical confirmation of metastasis. Postoperative radioactive iodine (I-131) therapy doses typically vary based on individual patient conditions (22); however, for patients identified as high-risk, an increased therapeutic dose may be considered. Furthermore, comprehensive genetic testing is recommended to identify actionable mutations for targeted therapies.

T stage, unilateral thyroid involvement, and TERT and BRAF V600E mutations were all independent predictors of lung metastasis. Advanced T stage (T3/T4; OR: 5.564, 95% CI: 1.769–17.502, P = 0.003) correlated with metastasis consistent with its role in local invasion (21, 23). Similarly, TERT promoter mutations, which promote uncontrolled growth by enhancing telomerase activity (24, 25), demonstrated strong predictive value (OR: 7.585, 95% CI: 1.245–46.199, P = 0.028), further confirming their established association with distant metastasis (26–29).

Although extensively implicated in thyroid cancer tumorigenesis and progression (30–33), the role of the BRAF V600E mutation in distant metastasis remains complex. Contrasting with its association with aggressive local features (34–37), our study identified the BRAF V600E as an independent factor inversely associated with lung metastasis risk (OR: 0.070, 95% CI: 0.022-0.225, P < 0.001). This inverse association is an intriguing phenomenon that finds support in other studies. Some research suggests that *BRAF* mutation may primarily influence local rather than distant metastasis (27, 38–40). For instance, Miguel Melo M et al. (27) reported that in PTC, compared to primary tumors, TERT promoter mutations increased while BRAF mutations decreased in metastatic lesions, indicating that BRAF may play a limited role during distant metastasis, whereas TERT mutations could be more critical. Similarly, Cheng X et al. (41) found a significantly higher frequency of *BRAF* mutations in PTC patients without distant metastases compared to those with metastases (84.8% vs. 51.5%, P < 0.0001), aligning with our observations.

The molecular mechanisms underlying this inverse association between BRAF mutation and lung metastasis remain unclear and warrant further investigation. One hypothesis is based on the concept of mutual exclusivity among oncogenic drivers, which has been reported in other studies (42, 43). We speculate that in this cohort of young patients with papillary thyroid carcinoma, the genetic drivers that result in distant metastasis may be other genes that are mutually exclusive with the BRAF V600E mutation. According to this hypothesis, when the BRAF V600E mutation is present, key genetic alterations required to complete the lung metastasis cascade may be absent or suppressed, potentially explaining the observed negative correlation. This mechanistic hypothesis remains speculative and requires direct validation through future studies.

However, the coexistence of BRAF and TERT mutations creates a powerful synergistic effect that profoundly enhances metastatic potential (44, 45). In our training cohort, the lung metastasis rate was 5.56% in patients harboring only the BRAF mutation, but this rate rose dramatically to 28.57% when both BRAF and TERT mutations were present. A similar pattern was observed in the validation cohort (**Supplementary Table 2**). This synergy can be explained by their mechanistic interaction. the BRAF-driven MAPK pathway can upregulate TERT promoter activity (46), while TERT cooperates with mutant BRAF to drive tumor dedifferentiation and progression through mechanisms such as the modulation of ribosomal biogenesis (47). Therefore, *BRAF* and *TERT* mutations may synergistically enhance metastasis.

Furthermore, our study characterized genetic mutations in a binary manner (presence/absence), without incorporating quantitative metrics such as variant allele frequency (VAF) or mutational burden. Emerging evidence suggests that VAF may reflect the clonal dominance of a mutation within the tumor, which could be associated with more aggressive behavior and poorer outcomes in thyroid cancer (48). Future studies incorporating VAF and other quantitative genetic features could provide a more nuanced understanding of tumor biology and further refine risk stratification models for lung metastasis in young patients with PTC.

We also conducted a comprehensive analysis of other genetic markers, including RAS family mutations (NRAS, HRAS, KRAS), TP53, and PIK3CA. As shown in **Table 2**, univariate analyses conducted within the training cohort confirmed that none of these mutations demonstrated a significant association with prognosis (RAS: OR = 1.488, P = 0.573; TP53: OR = 0.632, P = 0.676; PIK3CA: OR = 0.765, P = 0.809) and were therefore excluded from the final model. Future studies with larger sample sizes are needed to clearly evaluate the contribution of rare genetic events.

Not all regions or patients have access to genetic testing. Therefore, the clinical utility of this nomogram should be considered. We compared the predictive performance of the model using only two clinical variables (T stage and unilateral thyroid involvement) with that of the model utilizing all variables. As shown in **Supplementary Figure 2**, the model incorporating only clinical variables demonstrated reasonable predictive value, though its performance was markedly inferior to that of the full model integrating genetic data. Thus, to achieve optimal predictive accuracy, inclusion of BRAF and TERT mutation status is strongly recommended.

Although the 425-gene panel provides more genetic information compared to specific targeted gene testing, the 425-gene panel may prove difficult to implement widely in clinical practice due to its high cost. As our multivariate analysis revealed, only a subset of genes—specifically TERT and BRAF—along with clinical factors (T stage and unilateral thyroid involvement) were independently predictive of lung metastasis. This suggests that by detecting few specific genes(such as BRAF and *TERT* promoter mutation) and combining them with key clinical variables, we can predict lung metastasis, thereby reducing testing costs and enhancing feasibility.

There are some limitations to our study. First, as a retrospective analysis, it may be subject to selection bias during patient enrollment. Second, the model has only been validated internally. Although it demonstrates satisfactory performance within our cohort, external validation using independent, multi-institutional data is necessary to confirm its generalizability before clinical application, particularly regarding the cut-off value. We are actively collaborating with multiple leading cancer centers in China to establish an independent, multicenter cohort of young PTC patients for external validation, which will optimize the model and enhance its potential for translational application. Third, although other candidate genes such as *RAS* have been implicated in thyroid cancer metastasis (49), too few patients (only 3 with lung metastasis) presented with these mutations in our cohort to allow meaningful analysis. Larger samples are needed to evaluate other target genes and improve model accuracy. Fourth, we lacked data on patients’ thyroglobulin(Tg) levels. Tg serves as a core indicator for postoperative monitoring of differentiated thyroid cancer, demonstrating high sensitivity in detecting distant metastasis (50). Consequently, the absence of this data may diminish the practical value of this model. However, based on baseline genetic and pathological assessments, our model can identify young patients at high risk for lung metastasis during the early diagnostic stages—even before Tg levels change. Simultaneously, for specific patients with difficult-to-interpret Tg results (potentially affected by Tg antibodies), this model still provides supplementary predictive value to some extent. Therefore, we will integrate Tg data in the future to construct a more comprehensive predictive model and enhance its forecasting capabilities.

## Conclusion

We developed and validated a nomogram that integrates T stage, unilateral thyroid involvement, BRAF V600E, and TERT promoter mutations to individually predict the probability of lung metastasis in PTC patients under 55 years of age. This tool may assist clinicians in early risk stratification and personalized management.

## Data Availability

The original data are available from the corresponding author.
