# Supplementary material for: A Nomogram for Predicting Lung Metastasis in Papillary Thyroid Cancer Patients Aged Less Than 55 Years
Source: Front Endocrinol (Lausanne). 2025 Nov 11;16:1689674. doi: 10.3389/fendo.2025.1689674 (PMC12643860; doi:10.3389/fendo.2025.1689674)
Supplement: Supplementary file 1 [file Table1.docx]

Supplementary Material

# Supplementary Table3. List of included references of studies that compare index HPV tests and specimen with comparator assays and specimen by study design.

| Nr | Ref | Author | Journal | Year | Vol | Start Page | End Page | index test | Comparator test | Index specimen | Comparator specimen |
| --- | --- | --- | --- | --- | --- | --- | --- | --- | --- | --- | --- |
|  |  |  |  |  |  |  |  |  |  |  |  |
| **Study on the comparison of different detection methods** | | | | | | | | | | | |
| 1 | [1] | Ratnam | Clin Microbiol | 2010 | 48 | 2779 | 2785 | PreTect HPV-Proofer E6/E7 mRNA Assay | HC 2 | CS | CS |
| 2 | [2] | Hesselink | J Clin Microbiol | 2010 | 48 | 797 | 801 | PapilloCheck | GP5+/6+ EIA | CS | CS |
| 3 | [3] | Schopp | J Med Virol | 2010 | 82 | 605 | 615 | PapilloCheck | HC 2 | CS | CS |
| 4 | [4] | Kurian | Am J Clin Pathol | 2011 | 136 | 808 | 816 | Cervista | HC 2 | CS | CS |
| 5 | [5] | Clad | J Clin Microbiol | 2011 | 49 | 1071 | 1076 | APTIMA | HC 2 | CS | CS |
| 6 | [6] | Ratnam | J Clin Microbiol | 2011 | 49 | 557 | 564 | APTIMA | HC 2 | CS | CS |
| 7 | [7] | Wong | J Clin Virol | 2011 | 51 | 136 | 138 | Abbott | HC 2 | CS | CS |
| 8 | [8] | Park | J Clin Microbiol | 2012 | 50 | 2359 | 2365 | Abbott | HC 2 | CS | CS |
| 9 | [9] | Wong | J Clin Microbiol | 2012 | 50 | 25 | 29 | Cobas | HC 2 | CS | CS |
|  |  |  |  |  |  |  |  |  |  |  |  |
| 10 | [10] | Mesher | Eur J Cancer | 2013 | 49 | 2179 | 2186 | Abbott,The BD Onclarity,Cobas, APTIMA | HC 2 | CS | CS |
| 11 | [11] | Boers | J Clin Microbiol | 2014 | 52 | 4391 | 4393 | Cervista | HC 2 | CS | CS |
| 12 | [12] | Rebolj | Gynecol Oncol | 2014 | 135 | 474 | 480 | Cobas, APTIMA | HC 2 | CS | CS |
| 13 | [13] | Ronco | Lancet | 2014 | 383 | 524 | 532 | HC 2, GP5+/GP6+ PCR | LBC | CS | CS |
| 14 | [14] | Iftner | J Clin Microbiol | 2015 | 53 | 2509 | 2516 | APTIMA | HC 2 | CS | CS |
| 15 | [15] | Yu | J Med Virol | 2015 | 87 | 1587 | 1593 | Cobas | HC 2 | CS | CS |
| 16 | [16] | Ejegod | Papillomavirus Res | 2016 | 2 | 31 | 37 | BD Onclarity | HC 2 | CS | CS |
| 17 | [17] | Heard | J Clin Virol | 2016 | 81 | 6 | 11 | PapilloCheck | GP5+/6+ EIA | CS | CS |
| 18 | [18] | Larsson | Cell Oncol | 2017 | 40 | 521 | 527 | ddPCR | qPCR | CS | CS |
| 19 | [19] | Mao | ACS Appl Mater Interfaces | 2017 | 9 | 44307 | 44315 | Nanotechnology | PCR | CS | CS |
| 20 | [20] | Rohatensky | Bmc Cancer | 2018 | 18 |  |  | LAMP | PCR | Tissue block | Tissue block |
| 21 | [21] | Xu | Int J Mol Sc | 2018 | 19 | 2704 |  | INNO - LiPA | HC 2 | CS | CS |
| 22 | [22] | Bonde | J Clin Microbiol | 2020 | 58 | e01518-19 |  | BD Onclarity | HC 2 | CS | CS |
| 23 | [23] | Zhang | Infect Agents Cancer | 2020 | 15 | 65 |  | AmpFire,SeqHPV | Cobas | CS | CS |
| 24 | [24] | Avelino | Talanta | 2021 | 226 | 122118 |  | Nanotechnology | PCR | CS | CS |
| 25 | [25] | Malin | J Virol Methods | 2021 | 294 | 114193 |  | ddPCR | qPCR | CS | CS |
| 26 | [26] | Izadi | Anal Chim Acta | 2021 | 1187 |  |  | LAMP | qPCR | CS | CS |
| 27 | [27] | Andersen | Cancers | 2022 | 14 |  |  | NGS | Cobas | CS and SS | CS and SS |
| 28 | [28] | Mattox | Oral Oncol | 2022 | 128 | 105805 |  | ddPCR | qPCR | CS | CS |
| 29 | [29] | Giorgi Rossi | Int J Cancer | 2022 | 151 | 1047 | 1058 | APTIMA | HC 2 | CS | CS |
| 30 | [30] | Siravegna | Clin Cancer Res | 2022 | 28 | 719 | 727 | ddPCR | Histomorphological evaluation | Blood | Tissue |
| 31 | [31] | Zhan | Biosens Bioelectron | 2023 | 229 | 115229 |  | Nanotechnology | PCR | CS | CS |
| 32 | [32] | Han | J Clin Oncol | 2024 | 42 | 431 | 440 | NGS | ddPCR | Peripheral blood | Peripheral blood |
| 33 | [33] | Liu | Cell Mol Biol Lett | 2024 | 29 |  |  | RPA - CRISPR/Cas12a | qPCR | CS | CS |
| 34 | [34] | Pasquier | J Med Virol | 2024 | 96 |  |  | PacBio SMRT sequencing of E6/E7 | APTIMA,Sanger sequencing | CS | CS |
| 35 | [35] | White | Int J Cancer | 2024 | 154 | 53 | 64 | APTIMA | Cobas | CS | CS |
| 36 | [36] | Yin | Microbiol Spectr | 2024 |  |  |  | CRISPR/Cas12a | Cobas | CS | CS |
| **Study on the comparison of different test samples** | | | | | | | | | | | |
| 1 | [37] | Haguenoer | Br J Cancer | 2014 | 111 | 2187 | 2196 | PCR-based | PCR-based | SS | CS |
| 2 | [38] | Ducancelle | J Infect | 2015 | 71 | 377 | 384 | Abbott | Abbott | Urine | CS |
| 3 | [39] | Asciutto | Anticancer Res | 2017 | 37 | 4183 | 4187 | Cobas | Cobas | Urine and SS | CS |
| 4 | [40] | Gustavsson | Br J Cancer | 2018 | 118 | 896 | 904 | hpVIR | PAP smear cytology | SS | CS |
| 5 | [41] | Polman | Lancet Oncol | 2019 | 20 | 229 | 238 | GP5+/6+ PCR | GP5+/6+ PCR | SS | CS |
| 6 | [42] | Zheng | Mol Cancer | 2019 | 18 | 76 |  | miRNA sequencing, qRT - PCR and ddPCR | Pap smear and TCT | Plasma | CS |
| 7 | [43] | Lefeuvre | J Infect | 2020 | 81 | 248 | 254 | Anyplex | Anyplex | Urine | CS |
| 8 | [44] | Wang | J Mol Diagn | 2020 | 22 | 50 | 59 | ddPCR | ddPCR | Saliva | Tissue |
| 9 | [45] | Zhang | JAMA Netw Open | 2021 | 4 | e2140644 |  | NGS | Sanger | Menstrual blood(MB) | CS |
| 10 | [46] | Wulandari | Indones J Obstet Gynecol | 2023 | 11 | 161 | 165 | CerviScan | Cobas | Urine | CS |

CS: clinician taken cervical sample; SS: vaginal self-sample

# Supplementary Table 4. Characteristics of included studies evaluating HPV Assays for HPV related cancer screening.

| Evaluated assay | Study Design | Study(Author, year of publication and country) | Study population | Age range | Selection criteria | Criteria of outcome verification | Medium |
| --- | --- | --- | --- | --- | --- | --- | --- |
| PapilloCheck | Retrospective study | Schopp 2010 Germany and Denmark | Cervical smears from 881 women from two large cohort studies in Germany and Denmark, with 826 samples finally included in the analysis, of which 306 women had histological findings | Not  reported | Samples from two large cohort studies, including HC2-positive (87%) and HC2-negative groups ("problem" samples, such as abnormal cytology or colposcopy or HC2 results between 0.7 and 1.0 RLU/CO) | Histological analysis was performed on biopsies and/or cervical curettage samples from 306 women. | STM (Sample Transport Medium) |
|  | Retrospective study | Hesselink,  2010,  NL | Screening trial, arm  with conventional  cytology & GP5+/6+  PCR-EIA[60, 61] | 40 to 60 y | Cases: representative series of 192  women with CIN3+.  Controls: 1437 women without  CIN2+, negative cytology and no  cytological abnormalities within 8  years. | Colposcopy if HSIL+ is found. For hrHPV+ or ASCUS/LSIL, repeat cytology and HPV testing occurs at 6 and 18 months. At 6 months, colposcopy is performed if HSIL+ or ASCUS/LSIL with HPV+; at 18 months, if hrHPV+ or HSIL+. | PBS |
|  | Prospective Study | Heard,2016,France | A total of 1300 samples, including 1000 routine screening samples and 300 with cytological abnormalities from Scotland. of which 419 were from women under 30 and 881 from women aged 30 or older. 102 women had CIN2+ (55 with CIN3 or worse), and 747 had two consecutive cytology-negative smears. | 18 to 68 y | Samples were taken from the routine screening population and the cytological abnormalities population in Scotland, collected between August and October 2012 | Comparison of the relative accuracy of the PapilloCheck HPV assay versus the GP5+/6 + EIA for detecting CIN2+, using histological findings (CIN2+/CIN3+) as the primary outcome validation indicator | PreservCyt |
| Cervista | Prospective studies | Kurian,2011,USA | Patients who underwent cervical cytology at UMass Memorial Health Care from July 2009 to July 2010, a total of 601 ThinPrep Pap specimens, of which 533 cytology-negative cases were included in the analysis | 30 to 60 y | Serial collection of ThinPrep Pap specimens, irrespective of clinician's request for testing, analysed for cytology-negative cases | Sensitivity and specificity were calculated using biopsy results as the gold standard | PreservCyt |
|  | RCT | Boers,2014,Netherlands | 10,000 women from the SHENCCAST II study in Guangdong Province, China, and 510 cervical smear samples from women from the Netherlands participating in a routine national population-based cervical screening programme | 25 to 59 y(SHENCCAST II study) 30 to 60 y(Netherlands) | The SHENCCAST II study included 7,218 samples without and 109 with CIN2 lesions for non-inferiority analysis of Cervista vs. HC2 assays, along with 78 ASCUS and 31 NILM samples with confirmed CIN2 for relative sensitivity calculation. The Dutch sample was from a routine screening program | The relative clinical specificity and sensitivity of the Cervista HPV HR test was calculated using the HC2 test as a reference, and compliance with international guidelines was determined by non-inferiority testing | PreservCyt |
| PreTect HPV-Proofer assay | Cross-Sectional Study | Ratnam,2010,Canada | 1571 women with abnormal Pap cytology of any grade | 15 to 80 y | Women 15 years of age or older who had any grade of cytological abnormality within the previous 2 years and who had not received treatment or had not had a hysterectomy were eligible. | Histological diagnosis,with CIN2 as the disease endpoint and "gold standard." | PreservCyt |
| Aptima | Comparative diagnostic study | Clad,2011, Germany | 451 women with abnormal Pap screening result | Not  reported | Between February 2005 and May 2008,collected abnormal liquid-based cytology(LBC) and conventional cytology specimens in Universitaets-Frauenklinik Freiburg (Germany) | CIN2+ and CIN3+ | PreservCyt |
|  | Cross-sectional | Ratnam, 2011, Canada | 1418Referral cases: women referred for colposcopy 1373Routine screen: women undergoing routine screening | Referral group: 15 to 80 y  Routine screen: 16 to 81 y | Referral cases: newly diagnosed with abnormal Pap cytology or history of abnormal cytology and not treated Routine screen: normal Pap cytology screening | Histological diagnosis,with CIN2 as the disease endpoint | PreservCyt |
|  | Cohort Study | Mesher, 2013, UK | 1228 women referred with a borderline or single mildly dyskaryotic smear | 18 to 67 y | Women with one or more abnormal cervical smears and had not been previously treated for cervical intraepithelial neoplasia (CIN), no HPV testing as part of routine screening | The main endpoint for sensitivity was taken as CIN3+ (CIN2+ was also considered) Specificity is only reported for <CIN2 | PreservCyt |
|  | Prospective Cohort Study | Iftner,2015,Germany | 10,040 women aged 30 - 60 years undergoing routine cervical screening at three German centers | 30 to 60 y | Women without hysterectomy/destructive therapy of cervix, not pregnant, no abnormal cytology in past 6 months, not HIV-infected, not organ transplant recipients | Histological review based on colposcopy and biopsy (CIN nomenclature) | ThinPrep |
|  | Cohort Study | Rebolj,2014, Denmark | 367 consecutive women from Copenhagen with atypical squamous cells of undetermined significance or worse | 23 to 65 y | Women with ≥ASCUS cytology results | Worst histological diagnosis in 29 months from baseline | SurePath samples |
|  | Diagnostic Test Studies | Rossi, 2022, Italy | 41,127 women participating in routine HPV DNA screening | Not  reported | Women participating in routine HPV DNA screening | histopathological diagnosis of CIN2+ or CIN3+ | PreservCyt |
|  | Prospective cohort studies | White, 2024, Ireland | 10,150 women attending routine cervical screening (from 33 different primary care practices in Ireland) | 25 to 60 y | Recruited at the time of routine CervicalCheck primary screening smear; excluded pregnant women or those with a history of CIN2 + prior to enrollment | Taking histologically confirmed CIN2 + or higher grade lesions (CIN3 +) as the primary disease endpoint | PreservCyt |
| Abbott | Prospective observational study | Wong, 2011, China Hong Kong | 250 Asian women with ASC - US cytology samples from screening population in Hong Kong | 18 to 58 y | Diagnosed with ASC - US cytology | Colposcopic histology and/or repeat cytology | PreservCyt |
|  | Comparative study | Park,2012,Korea | 356 women who provided cervical swab specimens for cytologic exam | Not  reported | Cervical swab specimens were collected from women at Severance Hospital and Green Cross Reference Laboratory between August and October 2011 | Concordant results among the three assays were regarded as true-positive or -negative; results of genotyping and sequencing were considered true findings when the HPV assays presented discrepant results | PreservCyt |
|  | Cohort Study | Mesher, 2013, UK | 1228 women referred with a borderline or single mildly dyskaryotic smear | 18 to 67 y | Women with one or more abnormal cervical smears and had not been previously treated for cervical intraepithelial neoplasia (CIN), no HPV testing as part of routine screening | The main endpoint for sensitivity was taken as CIN3+ (CIN2+ was also considered) Specificity is only reported for <CIN2 | PreservCyt |
|  | Quasi - experimental Study | Ducancelle, 2015, France | Women aged 40 - 65 years who did not have a Pap smear over the past three years and did not respond to invitations for screening | 40 to 65 y | Women who did not respond to invitations for Pap smear screening from Cap Sante´ 49 | Cervical cytology analysis (according to Bethesda classification) and histology studies for women with cytological abnormalities | Abbott 4N73-04 |
| Cobas 4800 | Cross-Sectional Study | Wong, 2012, Canada | 466 women with cervical cytology specimens collected in PreservCyt for routine liquid - based cytology (LBC) | 15 to 60 y | Cervical specimens for HPV screening; subset of 472 specimens selected with preference for nonnormal cytology findings (HSIL, LSIL, ASCUS, NIL) | LA genotyping test for resolving discordant hc2 and c4800 results and confirming paired - positive results | PreservCyt |
|  | Cohort Study | Mesher, 2013, UK | 1228 women referred with a borderline or single mildly dyskaryotic smear | 18 to 67 y | Women with one or more abnormal cervical smears and had not been previously treated for cervical intraepithelial neoplasia (CIN), no HPV testing as part of routine screening | The main endpoint for sensitivity was taken as CIN3+ (CIN2+ was also considered) Specificity is only reported for <CIN2 | PreservCyt |
|  | Cohort Study | Rebolj, 2014, Denmark | 367 consecutive women from Copenhagen with atypical squamous cells of undetermined significance or worse | 23 to 65 y | Women with ≥ASCUS cytology results | Worst histological diagnosis in 29 months from baseline | SurePath samples |
|  | Cross-Sectional Study | Yu, 2015, South Korea | 861 women over 30 years of age | ≥30 y | 801 from Kangbuk Samsung Hospital Total Health Care Center and 60 with HSIL cytology results from Green Cross Reference Laboratory | Direct sequencing or LA HPV genotyping test for samples with discrepant results among the three HPV detection assays | PreservCyt |
| BD Onclarity | Case-control study | Ejegod,2016,Denmark | 269 women from the Copenhagen area, Denmark, referred for colposcopy due to cytological abnormalities and/or routine HPV positivity | 18 to 74 y | Women referred in line with national guidelines, excluding prior treatment for CIN, conization, LEEP, laser surgery, cryosurgery, known pregnancy, hysterectomy, cervical use of compounds within 24 hours prior to the study | Sensitivity, specificity and relative sensitivity and specificity of the three assays were calculated using the histological diagnostic results as the gold standard | BD Cervical Brush Dilution (CBD) |
|  | Diagnostic Test Studies | Bonde,2020,Denmark | Samples from 1295 women from the Danish cervical screening programme, including 998 consecutive screening samples and 297 enriched samples with cytological abnormalities (100 ASCUS, 100 LSIL, 97 HSIL) | 30 to 59 y | Women participating in the Danish cervical screening programme, with samples collected according to standard procedures, including the screening population and the enriched population with cytological abnormalities | The accuracy of the BD Onclarity assay versus the GP-EIA assay for ≥CIN2 and 2NILM was assessed by a non-inferiority test, using the histological diagnosis as the gold standard | SurePath |
| HPV test based on self-sampling (hpVIR) | RCT | Gustavsson, 2018, Sweden | 36390 women were randomised in two groups, one to perform self-sampling of VF for HPV test (n=17997, HPV arm) and the other group to perform screening by PAP smear cytology (n=18393, control arm) | 23 to 49y | Scheduled to participate in the organized screening program; aged 30 - 49 years old; no history of hysterectomy; no current pregnancy; no clinical test results related to cervical cancer within one year | Histological diagnosis of CIN2+ | Collected by self-sampling with a silicon brush and applied to the FTA elute card |
| INNO - LiPA HPV Genotyping Extra II | Diagnostic Test Validation Study | Xu, 2018, Belgium | 1600 consecutive cervical cell specimens | Not  reported | Women who participated in the Slovenian cervical cancer screening program | Histologically of CIN2 + and at least two consecutive cytological results of NILM | PreservCyt |
| GP5+/6+ PCR based on self-sampling | RCT | Polman, 2019, Netherlands | 187,473 women living in specific regions of the Netherlands, randomly divided into the self-sampling group | 29 to 61y | Received regular screening invitation; Signed informed consent; No previous hysterectomy, childbirth less than 6 months ago, and current pregnancy | Histologically of CIN2 + and CIN3 + | PreservCyt |
| Detecting specific miRNAs in exosomes by miRNA sequencing, qRT - PCR and ddPCR | Prospective Cohort Study | Zheng, 2019, China | 608 patients with cervical cancer and precancerous lesions; 98 patients' plasma samples for miRNA sequencing ; 46 new cervical cancer patients' cancer tissues and adjacent tissues for qRT - PCR analysis; 153 patients' plasma samples for ddPCR validation (validation set) | Average age was 50 ± 24 y | Patients with cervical cancer and precancerous lesions ; Patients with plasma samples also had corresponding clinical data; Tissue samples were from newly diagnosed cervical cancer patients | Taking histopathological test (biopsy result) as the diagnostic gold standard | PreservCyt |
| Urinary HPV testing (using Anyplex II HPV28 Detection technology) | Prospective Cohort Study | Lefeuvre, 2020, France | 13,535 women who had not had a Pap smear since 2010, with 1,915 ultimately included | 35 to 65 y | Received the invitation letter; Signed the informed consent form; No history of hysterectomy, no recent Pap smear, did not refuse to participate in the study, resided within the department, not deceased, complete file, not hospitalized, and with clear reasons | Cervical cytology examination and histological examination | Stored in a sterile container |
| ddPCR | Experimental Study | Larsson,2017,Sweden | HPV-positive patient samples from Örebro University Hospital, including cervical LBC samples and FFPE samples | Not  reported | Samples were selected based on HPV 16, 18, 33 or 45 positivity, covering both high and low viral load samples and representing different types of samples | Evaluation of ddPCR by analysing viral load in different samples; comparison of ddPCR and qPCR data for HPV 16; validation of the accuracy of the assay with known HPV copies in cell lines | PreservCyt |
|  | Retrospective Cohort Study | Borkowska,2021,Poland | 113 patients with surgically treated HNSCC | 42 to 91 y | Diagnosed with HNSCC and surgically treated | Histopathological diagnosis | FFPE |
|  | Prospective Cohort Study | Siravegna, 2022, USA | 140 subjects, including 70 cases with newly diagnosed or suspected HPV + HNSCC and untreated, and 70 HPV negative HNSCC patients and non-cancer controls | 44 to 84 y | Newly diagnosed or suspected HPV + HNSCC and untreated, willing to provide blood samples and sign informed consent | Histomorphological assessment and direct HPV testing (HPV DNA polymerase chain reaction or RNA in situ hybridisation) as the gold standard | Blood samples |
|  | Cohort Study | Malin 2021, Sweden | A total of 209 cervical cancer patients from Örebro University Hospital were identified, including 17 with multiple high-risk HPV genotypes and 145 with single-genotype infections. The samples represented various histological subtypes: 128 squamous cell carcinomas, 15 adenocarcinomas, and others. FIGO stages included 33 stage I, 86 stage II, 21 stage III, and 8 stage IV | 24 to 91 y | The first part includes 25 HPV-positive samples from Örebro University Hospital (2011-2017), selected by sample type (FFPE tissue and liquid cytology) and HPV genotypes. The second part includes cervical cancer patients (1993-2014) with FFPE tumor samples containing multiple or single high-risk HPV genotypes | The viral load (VL) was calculated from the ddPCR results, and the analysis was validated by comparing the differences in VL between different groups | PreservCyt |
| NGS | Cohort Study | Zhang, 2021, China | 120 premenopausal women who were positive for hrHPV by cervical HPV testing | 20 to 52 y | Premenopausal women; Positive for hrHPV by cervical HPV testing; Agreed to participate and provided signed informed consent; No refusal to participate, no menstruation within 3 months, no small menstrual bloodstain area on sanitary pads, etc. | Sanger sequencing was used as the criterion standard for detecting hrHPV genotypes | Menstrual blood samples |
|  | Diagnostic Test Studies | Andersen, 2022, Denmark | 93 pairs (CS and SS) from women diagnosed with ASC-US and reflex HPV tested in the Danish cervical cancer screening program | 30 to 59 y | Selected from a specific cohort: Samples are GP-collected cervical samples and corresponding cervico-vaginal self-samples; Women diagnosed with ASC-US and reflex HPV tested; Previously tested with Cobas 4800 and CLART HPV4S assays | Using the "consensus" result of the NGS, Cobas 4800, and CLART HPV4S assays as the reference standard | DNA Blood Collection Tube |
|  | Prospective Cohort Study | Han,2023,Canada | 84 patients with stage IB2 - IVA cervical cancer who were enrolled and received definitive chemoradiation (CRT) (finally 75 patients were evaluable) | ≥18y | Female patients with histologically confirmed 2009 International Federation of Gynecology and Obstetrics (FIGO) stage IB2 - IVA cervical squamous cell carcinoma, adenocarcinoma, or adenosquamous carcinoma and planned for definitive CRT | The primary endpoint was progression - free survival | Streck Cell - Free DNA BCT tubes |
|  | Diagnostic Test Studies | Pasquier, 2024, France | 30 biopsy samples that had been tested for high - and low - risk HPV by L1 PCR and Sanger sequencing (from various sources such as laryngeal, genital, cutaneous, anal, and esophageal) and 281 routine cervical samples collected in PreservCyt that tested positive for high - risk HPV E6/E7 mRNA by APTIMA HPV assay | Not  reported | Biopsy samples were previously tested and stored samples; cervical samples were routine screening leftover samples collected in PreservCyt and positive for high - risk HPV E6/E7 mRNA by APTIMA HPV assay | Using L1 Sanger sequencing and APTIMA HPV assay as controls, verifying the performance of PacBio E6/E7 SMRT sequencing by comparing the consistency, sensitivity. | PreservCyt |
| CRISPR/Cas12a | Diagnostic Test Studies | Liu,2024,China | Mainly 34 clinical cervical fluid samples sourced from the gynecology outpatient clinic and laboratory department of the hospital | Not  reported | Clinical cervical fluid samples were collected routinely in the hospital; pathogen - related samples were used for specificity test validation | Using the fluorescent quantitative PCR (QPCR) detection method as a control, comparing the consistency, sensitivity, and specificity of HPV detection in clinical samples between the two methods to verify the reliability of the H - MRC12a detection system | NA |
|  | Diagnostic Test Studies | Yin, 2024, China | 258 clinical cervical swab samples obtained from Xiangyang Central Hospital | Not  reported | The clinical samples were obtained from the hospital and approved by the ethics committee (2022 - 089). Both the commercial qPCR kit (Cobas HPV test) and the established CRISPR/Cas12a detection platform in this study were used to test the samples. | Clinical sensitivity and specificity were evaluated by comparing the detection results of the CRISPR/Cas12a-based assay with those of the Cobas HPV test on the 258 clinical samples. | NA |
| Liquid biopsy | Case-control study | Asciutto, 2017, Sweden | 218 women with abnormal cervical smear in screening program or with symptoms were invited | 19 to 71 y | Women with abnormal cervical smear in screening program or with symptoms were invited. Self-collected vaginal, urine and clinician-taken cervical samples were analyzed and compared | Histopathological review when available, otherwise cytology. HPV analysis results from all samples. | PreservCyt |
|  | Experimental Study | Wang,2020, multiple countries (USA, China, UK etc.) | 10 HPV - OPC patients and healthy volunteers | Not  reported | HPV - OPC patients recruited from the multicenter De - Escalate trial; healthy volunteers with instructions to refrain from eating, drinking, smoking, or oral hygiene procedures for at least 1 hour before saliva collection | HPV16 DNA detection in exosomes from patients' saliva, comparing with tissues/biopsies positive for HPV16 | Saliva |
|  | Diagnostic Test Studies | Wulandari, 2023, Indonesia | 876 adult women who completed the examination and visited for routine physical examination or cervical cancer screening | 20 to 50 y | Sexually active adult women who visited for routine physical examination or cervical cancer screening | Taking the cervical swab HPV - DNA test based on the Cobas 6800 system as the gold standard | Urine stored in a sterile container,cervical swab stored in ThinPrep PreservCyt Solution |
| Nanotechnology | Diagnostic Test Study | Mao,2017,China | Cervical smear samples from 52 women | ≥20 y | Cervical scraping samples from women undergoing colposcopic cervical biopsy were selected and included different cytological types such as ASCUS, LSIL, HSIL and SCC | The sensitivity, specificity and consistency of the Cys - Sso7d/Au NP probe for the detection of clinical samples were assessed by calculating the sensitivity, specificity and consistency of the Cys - Sso7d/Au NP probe using PCR as the gold standard | NA |
|  | Experimental Study | Avelino,2021,Brazil | Clinical samples from women infected with HPV | Not  reported | Samples containing specific HPV genotypes and cDNA samples from HPV-infected cervical specimens | The performance of the biosensor was verified using electrochemical techniques and atomic force microscopy for structural analysis | PBS |
| LAMP | Experimental Study | Rohatensky ,2018,Canada | Human cell lines and clinical samples from patients with oropharyngeal squamous cell carcinoma | Not  reported | Cell lines and plasmids known to contain specific HPV subtypes were selected for the study. | Confirmation of positive LAMP reaction results by agarose gel electrophoresis, turbidimetry and optical density measurements; comparison of the performance of the LAMP assay using the PCR assay as a reference | NA |
|  | Clinical Sample Validation Study | Izadi,2021, Czech Republic | 19 cervical samples from uninfected women and women infected with HPV 16 or HPV 18 genotypes causing precancerous cervical lesions. | 20 to 56 y | Clinical samples were obtained from patients referred to the Obstetrics and Gynaecology Clinic of the University Hospital of Brno, approved by the Ethics Committee and with informed consent from the patients | Evaluation of assay performance using classical PCR as the gold standard. Use of the INNO - LiPA HPV Genotyping EXTRA II commercial kit to assist in assessing HPV status | Homogenization buffer (HB) |
| AmpFire,SeqHPV | Diagnostic Test Study | Zhang,2020,China | 6042 women who had not been screened for cervical cancer 3 years previously, were non-pregnant, had an intact uterus and had no history of pelvic radiotherapy | 30 to 55 y | Eligible for age, screening history, pregnancy, and uterine and pelvic radiotherapy; samples from women participating in the China Multicentre Screening Trial (CHIMUST) in selected regions | Histological findings (normal, CIN1, CIN2, CIN3/AIS or cancer) as the gold standard | PreservCyt |

PBS: phosphate-buffered saline

**References**

1. Ratnam, S., et al., Clinical Performance of the PreTect HPV-Proofer E6/E7 mRNA Assay in Comparison with That of the Hybrid Capture 2 Test for Identification of Women at Risk of Cervical Cancer. Journal of Clinical Microbiology, 2010. **48**(8): p. 2779-2785.

2. Hesselink, A.T., et al., Comparison of the Clinical Performance of PapilloCheck Human Papillomavirus Detection with That of the GP5+/6+-PCR-Enzyme Immunoassay in Population-Based Cervical Screening. Journal of Clinical Microbiology, 2010. **48**(3): p. 797-801.

3. Schopp, B., et al., Evaluation of the performance of the novel PapilloCheck HPV genotyping test by comparison with two other genotyping systems and the HC2 test. J Med Virol, 2010. **82**(4): p. 605-15.

4. Kurian, E.M., et al., Cervista HR and HPV 16/18 Assays vs Hybrid Capture 2 Assay Outcome Comparison in Women With Negative Cervical Cytology. American Journal of Clinical Pathology, 2011. **136**(5): p. 808-816.

5. Clad, A., et al., Performance of the Aptima High-Risk Human Papillomavirus mRNA Assay in a Referral Population in Comparison with Hybrid Capture 2 and Cytology. Journal of Clinical Microbiology, 2011. **49**(3): p. 1071-1076.

6. Ratnam, S., et al., Aptima HPV E6/E7 mRNA Test Is as Sensitive as Hybrid Capture 2 Assay but More Specific at Detecting Cervical Precancer and Cancer. Journal of Clinical Microbiology, 2011. **49**(2): p. 557-564.

7. Wong, O.G.W., et al., Efficacy of Abbott RealTime High Risk HPV test in evaluation of atypical squamous cells of undetermined significance from an Asian screening population. Journal of Clinical Virology, 2011. **51**(2): p. 136-138.

8. Park, Y., et al., Comparison of the Abbott RealTi<i>m</i>e High-Risk Human Papillomavirus (HPV), Roche Cobas HPV, and Hybrid Capture 2 Assays to Direct Sequencing and Genotyping of HPV DNA. Journal of Clinical Microbiology, 2012. **50**(7): p. 2359-2365.

9. Wong, A.A., et al., Comparison of the Hybrid Capture 2 and cobas 4800 Tests for Detection of High-Risk Human Papillomavirus in Specimens Collected in PreservCyt Medium. Journal of Clinical Microbiology, 2012. **50**(1): p. 25-29.

10. Mesher, D., et al., Comparison of human papillomavirus testing strategies for triage of women referred with low-grade cytological abnormalities. European Journal of Cancer, 2013. **49**(9): p. 2179-2186.

11. Boers, A., et al., Clinical Validation of the Cervista HPV HR Test According to the International Guidelines for Human Papillomavirus Test Requirements for Cervical Cancer Screening. Journal of Clinical Microbiology, 2014. **52**(12): p. 4391-4393.

12. Rebolj, M., et al., Comparison of three human papillomavirus DNA assays and one mRNA assay in women with abnormal cytology. Gynecologic Oncology, 2014. **135**(3): p. 474-480.

13. Ronco, G., et al., Efficacy of HPV-based screening for prevention of invasive cervical cancer: follow-up of four European randomised controlled trials. Lancet, 2014. **383**(9916): p. 524-532.

14. Iftner, T., et al., Head-to-Head Comparison of the RNA-Based Aptima Human Papillomavirus (HPV) Assay and the DNA-Based Hybrid Capture 2 HPV Test in a Routine Screening Population of Women Aged 30 to 60 Years in Germany. Journal of Clinical Microbiology, 2015. **53**(8): p. 2509-2516.

15. Yu, S., et al., Comparison of clinical performances among Roche Cobas HPV, RFMP HPV PapilloTyper and Hybrid Capture 2 assays for detection of high-risk types of human papillomavirus. Journal of Medical Virology, 2015. **87**(9): p. 1587-1593.

16. Ejegod, D.M., et al., Clinical and analytical performance of the BD Onclarity™ HPV assay for detection of CIN2+ lesions on SurePath samples. Papillomavirus Res, 2016. **2**: p. 31-37.

17. Heard, I., et al., Clinical and analytical performance of the PapilloCheck HPV-Screening assay using the VALGENT framework. J Clin Virol, 2016. **81**: p. 6-11.

18. Lillsunde Larsson, G. and G. Helenius, Digital droplet PCR (ddPCR) for the detection and quantification of HPV 16, 18, 33 and 45 - a short report. Cell Oncol (Dordr), 2017. **40**(5): p. 521-527.

19. Mao, J.Y., et al., DNA Modulates the Interaction of Genetically Engineered DNA-Binding Proteins and Gold Nanoparticles: Diagnosis of High-Risk HPV Infection. ACS Appl Mater Interfaces, 2017. **9**(51): p. 44307-44315.

20. Rohatensky, M.G., et al., Assessing the performance of a Loop Mediated Isothermal Amplification (LAMP) assay for the detection and subtyping of high-risk suptypes of Human Papilloma Virus (HPV) for Oropharyngeal Squamous Cell Carcinoma (OPSCC) without DNA purification. Bmc Cancer, 2018. **18**.

21. Xu, L., et al., Clinical Evaluation of INNO-LiPA HPV Genotyping <i>EXTRA</i> II Assay Using the VALGENT Framework. International Journal of Molecular Sciences, 2018. **19**(9).

22. Bonde, J.H., et al., Clinical and Analytical Performance of the BD Onclarity HPV Assay with SurePath Screening Samples from the Danish Cervical Screening Program Using the VALGENT Framework. Journal of Clinical Microbiology, 2020. **58**(2).

23. Zhang, W., et al., Evaluation of an isothermal amplification HPV detection assay for primary cervical cancer screening. Infectious Agents and Cancer, 2020. **15**(1).

24. Avelino, K., et al., Flexible sensor based on conducting polymer and gold nanoparticles for electrochemical screening of HPV families in cervical specimens. Talanta, 2021. **226**: p. 122118.

25. Malin, K., et al., Optimization of droplet digital PCR assays for the type-specific detection and quantification of five HPV genotypes, including additional data on viral loads of nine different HPV genotypes in cervical carcinomas. J Virol Methods, 2021. **294**: p. 114193.

26. Izadi, N., et al., Electrochemical bioassay coupled to LAMP reaction for determination of high-risk HPV infection in crude lysates. Analytica Chimica Acta, 2021. **1187**.

27. Andersen, K., et al., Targeted Next Generation Sequencing for Human Papillomavirus Genotyping in Cervical Liquid-Based Cytology Samples. Cancers, 2022. **14**(3).

28. Mattox, A.K., et al., Comparison of next generation sequencing, droplet digital PCR, and quantitative real-time PCR for the earlier detection and quantification of HPV in HPV-positive oropharyngeal cancer. Oral Oncol, 2022. **128**: p. 105805.

29. Giorgi Rossi, P., et al., Performance of HPV E6/E7 mRNA assay as primary screening test: Results from the NTCC2 trial. Int J Cancer, 2022. **151**(7): p. 1047-1058.

30. Siravegna, G., et al., Cell-Free HPV DNA Provides an Accurate and Rapid Diagnosis of HPV-Associated Head and Neck Cancer. Clinical Cancer Research, 2022. **28**(4): p. 719-727.

31. Zhan, X., et al., DNA tetrahedron-based CRISPR bioassay for treble-self-amplified and multiplex HPV-DNA detection with elemental tagging. Biosens Bioelectron, 2023. **229**: p. 115229.

32. Han, K., et al., Clinical Validation of Human Papilloma Virus Circulating Tumor DNA for Early Detection of Residual Disease After Chemoradiation in Cervical Cancer. Journal of Clinical Oncology, 2024. **42**(4).

33. Liu, Y., et al., A multiplex RPA-CRISPR/Cas12a-based POCT technique and its application in human papillomavirus (HPV) typing assay. Cellular & Molecular Biology Letters, 2024. **29**(1).

34. Pasquier, C., et al., HPV genotyping in clinical samples using long-read single-molecule real-time sequencing. Journal of Medical Virology, 2024. **96**(5).

35. White, C., et al., Performance of the HPV E6/E7 mRNA Aptima HPV assay combined with partial genotyping compared with the HPV DNA Cobas 4800 HPV test for use in primary screening: Results from the CERVIVA HPV primary screening study in Ireland. International Journal of Cancer, 2024. **154**(1): p. 53-64.

36. Yin, L., et al., Development and evaluation of a CRISPR/Cas12a-based diagnostic test for rapid detection and genotyping of HR-HPV in clinical specimens. Microbiology Spectrum, 2024.

37. Haguenoer, K., et al., Vaginal self-sampling is a cost-effective way to increase participation in a cervical cancer screening programme: a randomised trial. British Journal of Cancer, 2014. **111**(11): p. 2187-2196.

38. Ducancelle, A., et al., Home-based urinary HPV DNA testing in women who do not attend cervical cancer screening clinics. Journal of Infection, 2015. **71**(3): p. 377-384.

39. Asciutto, K.C., et al., Vaginal and urine self-sampling compared to cervical sampling for HPV-testing with the cobas 4800 HPV test. Anticancer Research, 2017. **37**(8): p. 4183-4187.

40. Gustavsson, I., et al., Randomised study shows that repeated self-sampling and HPV test has more than twofold higher detection rate of women with CIN2+histology than Pap smear cytology. British Journal of Cancer, 2018. **118**(6): p. 896-904.

41. Polman, N.J., et al., Performance of human papillomavirus testing on self-collected versus clinician-collected samples for the detection of cervical intraepithelial neoplasia of grade 2 or worse: a randomised, paired screen-positive, non-inferiority trial. Lancet Oncol, 2019. **20**(2): p. 229-238.

42. Zheng, M., et al., Exosomal let-7d-3p and miR-30d-5p as diagnostic biomarkers for non-invasive screening of cervical cancer and its precursors. Mol Cancer, 2019. **18**(1): p. 76.

43. Lefeuvre, C., et al., Urinary HPV DNA testing as a tool for cervical cancer screening in women who are reluctant to have a Pap smear in France. Journal of Infection, 2020. **81**(2): p. 248-254.

44. Wang, Z.Y., et al., Acoustofluidic Salivary Exosome Isolation <i>A Liquid Biopsy Compatible Approach for Human Papillomavirus</i>-<i>Associated Oropharyngeal Cancer Detection</i>. Journal of Molecular Diagnostics, 2020. **22**(1): p. 50-59.

45. Zhang, J., et al., Feasibility and Accuracy of Menstrual Blood Testing for High-risk Human Papillomavirus Detection With Capture Sequencing. Jama Network Open, 2021. **4**(12).

46. Wulandari, D., et al., Diagnostic Performance of Urine-based HPV-DNA Test (CerviScan, Bio Farma) as Cervical Cancer Screening Tool in Adult Women. Indonesian Journal of Obstetrics and Gynecology, 2023. **11**(3).
